# Psychosocial Adjustment to Illness among HIV-Positive People from Buenos Aires, Argentina*

**DOI:** 10.17533/udea.iee.v40n1e11

**Published:** 2022-03-30

**Authors:** Carlos Jesús Canova Barrios

**Affiliations:** 1 Nurse, Ph.D in Medical Sciences. Professor, Universidad de Ciencias Empresariales y Sociales, Buenos Aires (Argentina). Email: Carlos.canova1993@gmail.com Universidad de Ciencias Empresariales y Sociales Universidad de Ciencias Empresariales y Sociales Buenos Aires Argentina Carlos.canova1993@gmail.com

**Keywords:** psychosocial support systems, HIV infections, HIV-1, treatment adherence and compliance, social adjustment., sistemas de apoyo psicosocial, Infecciones por VIH, VIH-1, cumplimiento y adherencia al tratamiento, Ajuste social., sistemas de apoio psicossocial, infections à VIH, HIV-1, cooperação e adesão ao tratamento, ajustamento social.

## Abstract

**Objective.:**

To analyze the process of psychosocial adjustment to illness in a sample of people living with the Human Immunodeficiency Virus from Buenos Aires, Argentina.

**Methods.:**

Cross-sectional analytical study. The sample consisted of 144 HIV-positive people chosen by simple random sampling. The PAIS-SR questionnaire was used to measure the Psychosocial Adjustment process, which is made up of 46 items organized into 7 domains, whose final score ranges between 0 and 100, interpreted so that the higher the score, the worse the psychosocial adjustment process.

**Results.:**

The respondents reported were mostly male (82.63%), single (61.80%), with university studies (50.00%), without children (74.30%), and with a steady job (88.19%); the mean age of the participants was 43.8 years. The median global score was 51.4 (IQR: 12). The domains with the worst perception of psychosocial adjustment were: Health care orientation (Me: 56, IQR: 20), extended family relationship (Me: 55, IQR: 20), and Sexual relationship (Me: 54, IQR: 14), while those who had a better perception of adjustment were: Domestic environment (Me: 48, IQR: 8), Psychological distress (Me: 48, IQR: 17), Social environment (Me: 50, IQR: 18) and Vocational environment (Me: 50, IQR: 12). It was found that patients with a poor psychosocial adjustment process had low adherence to treatment, higher frequency of smoking, and sedentary lifestyle (*p*<0.001), while male sex, older age, and employment were related to a better psychosocial adjustment process (*p*<0.001).

**Conclusion.:**

The process of psychosocial adjustment to illness in the study group is medium; adjustment was positively related to self-care habits such as better adherence to pharmacological treatment, physical activity, and not smoking.

## Resumen

**Objetivo.:**

Analizar el proceso de ajuste psicosocial en una muestra de personas que conviven con el Virus de la Inmunodeficiencia Humana de la Ciudad Autónoma de Buenos Aires, Argentina.

**Métodos.:**

Estudio analítico de corte transversal. La muestra estuvo integrada por 144 personas HIV-positivas elegidos mediante muestreo aleatorio simple. Se empleó para la medición del proceso de Ajuste Psicosocial el cuestionario PAIS-SR, el cual está integrado por 46 ítems organizados en 7 dominios, cuyo puntaje final oscila entre 0 y 100, interpretándose de modo que, a mayor puntaje es peor el proceso de ajuste psicosocial.

**Resultados.:**

Los encuestados fueron en su mayoría sexo masculino (82.63%), solteros (61.80%), con estudios universitarios (50%), sin hijos (74.30%) y con trabajo estable (88.19%); la media de edad de los participantes fue de 43.8 años. La mediana del puntaje global fue de 51.4 (RIQ:12). Los dominios con peor precepción de ajuste psicosocial fueron: Orientación al cuidado de la salud (Me: 56, RIQ: 20), Relación con la Familia Extendida (Me: 55, RIQ: 20) y Relaciones sexuales (Me: 54, RIQ: 14), mientras que los que tuvieron mejor percepción de ajuste fueron: Ambiente familiar (Me: 48, RIQ: 8), Distrés psicológico (Me: 48, RIQ: 17), Ambiente social (Me: 50, RIQ: 18) y Ambiente Laboral (Me: 50, RIQ: 12). Se encontró que los pacientes con un mal proceso de ajuste psicosocial presentaban una baja adherencia al tratamiento y más frecuencia de tabaquismo y sedentarismo (*p*<0.001), mientras que un mejor proceso de ajuste psicosocial se asoció a ser de sexo masculino, mayor edad y tener empleo estable (*p*<0.001).

**Conclusión.:**

El proceso de ajuste psicosocial a la enfermedad en el grupo de estudio es medio; el ajuste se relacionó en forma positiva con hábitos de autocuidado como una mejor adherencia al tratamiento farmacológico, realización de actividad física y no fumar.

## Resumo

**Objetivo.:**

Analisar o processo de ajuste psicossocial numa amostra de pessoas que convivem com o Vírus da Imunodeficiência Humana da Cidade Autônoma de Buenos Aires, Argentina.

**Métodos.:**

Estudo analítico de corte transversal. A amostra esteve integrada por 144 pessoas HIV-positivas elegidos mediante amostragem aleatório simples. Se empregou para a medição do processo de Ajuste Psicossocial o questionário PAIS-SR, o qual está integrado por 46 itens organizados em 7 domínios, cuja pontuação final oscila entre 0 e 100, interpretando-se de modo que, a maior pontuação é pior que o processo de ajuste psicossocial.

**Resultados.:**

Os entrevistados foram na sua maioria do sexo masculino (82.63%), solteiros (61.80%), com estudos universitários (50%), sem filhos (74.30%) e com trabalho estável (88.19%); a média de idade dos participantes foi de 43 anos. A média da pontuação global foi de 51.4 (RIQ:12). Os domínios com pior percepção de ajuste psicossocial foram: Orientação ao cuidado da saúde (Me: 56, RIQ: 20), Relação com a Família Estendida (Me: 55, RIQ: 20) e Relações sexuais (Me: 54, RIQ: 14), enquanto que os que tiveram melhor percepção de ajuste foram: Ambiente familiar (Me: 48, RIQ: 8), Distresse psicológico (Me: 48, RIQ: 17), Ambiente social (Me: 50, RIQ: 18) e Ambiente Laboral (Me: 50, RIQ: 12). Se encontrou que os pacientes com um mal processo de ajuste psicossocial apresentavam uma baixa aderência ao tratamento e mais frequência de tabaquismo e sedentarismo (*p*<0.001), enquanto um melhor processo de ajuste social se associou a ser de sexo masculino, maior idade e posse de emprego se relacionaram com um melhor processo de ajuste psicossocial (*p*<0.001).

**Conclusão.:**

O processo de ajuste psicossocial à doença no grupo de estudo é médio; o ajuste se relacionou em forma positiva com hábitos de autocuidado como uma melhor aderência ao tratamento farmacológico, realização de atividade física e não fumar.

## Introduction

Today, Human Immunodeficiency Virus (HIV) continues to be a major global public health concern. There are approximately 38 million people currently living with HIV and 33 million people have died due to HIV-related causes.(1) In Argentina, according to the Ministry of Health, 139,000 people living with HIV, of which 83% know their condition. In 2018, 5,800 people were diagnosed with the infection (36.5% of them in advanced stages), the perinatal transmission rate was 4.6%, and 1,458 people died from causes related to Acquired Immunodeficiency Syndrome (AIDS).(2)

The progression of HIV infection leads to a deterioration of immune response, leading to AIDS.(3) Conditions such as social marginalization and discrimination generate high levels of stress, causing the appearance of various psychophysical pathologies, which leads to considering HIV infection as a psychosocial problem,(4-6) leading to a new variable of analysis and interest: the Psychosocial Adjustment Process. This process includes aspects such as health care orientation, vocational environment, domestic environment, sexual relationship, extended family relationship, social environment, and psychological distress. All of them have an impact on the health maintenance of HIV-positive patients, directly influence on the implementation of self-care behaviors or in its deficit (when support is lacking), low adherence to treatment, and other crucial aspects for the disease management.(7-9) In this regard, authors such as Villacres-García et al,(10) mention that the lack of economic resources to comply with antiretroviral therapy and medical check-ups, low self-esteem, physical abuse, and discrimination, entail low adherence to treatment and, consequently, a decrease in the CD4 count and higher viral load. Therefore, the measurement and approach to psychosocial well-being are considered of great importance in the comprehensive approach of the subject, separating the biologists and reductionist conception of the treatment of disease, only based on the improvement of clinical laboratory parameters to advance on the inclusion of psychosocial aspects such as the promotion of healthy family and work relationships, the promotion of self-esteem and the reduction of distress and anguish.

For nurses who are part of the HIV multidisciplinary teams in HIV specialized centers and primary care areas, and based on the above, the need for a holistic and comprehensive approach to people living with HIV is evident. This approach requires not only knowledge about the virus and its associated disease, but also the development of skills that allow them to provide optimal care. The inclusion of the family as an essential part of care, the recognition of education as a tool for the reduction of discrimination and a potential strategy for social inclusion, the promotion of self-care including treatment adherence, inter alia, are some of the relevant aspects that are often neglected in the caring role. According to Evans & Dukes,(11) it is necessary to delimit and strengthen the roles, influences, and responsibilities of nurses in the approach to the HIV patient, which includes the incorporation of more professionals in community areas, hospitals, educational and research services, as well as the participation and in the execution of activities of prevention, testing, disclosure, promotion of treatment adherence, family education, and approach of people with HIV/AIDS-associated disorders during hospitalization. The objective of this study was to analyze the process of psychosocial adjustment to illness in a sample of people living with the Human Immunodeficiency Virus from Buenos Aires, Argentina.

## Methods

Study design. A cross-sectional study was conducted in the first half of 2020.

Population and sample. The population consisted of HIV-positive patients from Buenos Aires, and the sample was selected from private clinics in the city. The sample calculation sought to ensure a confidence level of 95%, the statistical power of 80% and, a proportion of 20%.(12,13) The sampling technique was simple random sampling. The sample was selected using the medical appointment schedule, which were loaded into an Excel spreadsheet and then the patients were randomized and selected using the Work in Epidemiology tool (WinEpi©2006). Twelve patients were surveyed per week for a period of 3 months (13 weeks). The sample to recruit was 121 people and anticipating a 20% loss rate (incomplete or poorly filled out instruments), 153 individuals were recruited. The final sample consisted of 144 observations (9 incomplete records were eliminated).

Inclusion and exclusion criteria. Individuals who had been diagnosed with Human Immunodeficiency Virus Infection were included within these criteria, aged between 18 and 60 years, with no altered mental status (diagnosis of dementia or cognitive impairment), with more than 6 months of diagnosis and more than 3 months in antiretroviral treatment, and who did not have any degree of disability (visual, auditory, motor or mental), and were excluded people with hormone replacement therapy, those who have not completed the questionnaire, have a history of hospitalization in the last 30 days and pregnant women.

Instruments. For data collection, the Psychosocial Adjustment to Illness Scale - Self Report (PAIS-SR) was implemented in its Spanish validated version (Cronbach's alpha 0.93).(14-16) The instrument was licensed for use by Clinical Psychometric Research, Inc.

This instrument is made up of 46 items aimed to assess both the psychological and social adjustment of medical patients in various clinically important/ relevant domains applicable to a wide spectrum of chronic disorders.(14) The construct measures its items in 7 specific domains: health care orientation (items 1-8), vocational environment (items 9-14), domestic environment (items 15-22), sexual relationships (items 23-28), extended family relationships (items 29-33), social environment (items 34-39), and psychological distress (items 40-46).

Regarding the interpretation of the PAIS-SR scores, the global score indicates the general psychosocial adjustment to illness of the patient, while the domains and the items scores can help with the assessment of specific areas of psychosocial adjustment. An overall score above 62 points indicates that the patient has difficulty adapting to the disease. Higher scores indicate a lower level of adjustment. Each item of PAIS-SR is rated on a 4-point scale that is scored in an increasing sense (odd-numbered questions) or decreasing (even-numbered questions) to which numerical values ranging from 0 to 3 are assigned, and which are then grouped into the 7 domains mentioned as indicated in the PAIS-SR administration, scoring, and analysis procedure manual.

The score in the health care orientation domain ranges between 0 and 24, vocational environment between 0 and 18 points, domestic environment between 0 and 24 points, sexual relationship between 0 and 18 points, extended family relationships between 0 and 15 points, social environment between 0 and 18 points, and psychological distress between 0 and 21 points. These raw scores are then converted to T-scores ranging from 0 to 100.

The above information was complemented with a battery of questions that sought to investigate the sociodemographic profile of the respondent, self-care habits (smoking and physical activity), and the 4-item Morisky Medication Adherence Scale to determine the adherence to pharmacological treatment.(17) The latter is made up of 4 dichotomous response items (yes or no) that assess the barriers to correct therapeutic adherence: Do you ever forget to take your medications? At times, are you not careful about taking your medicine? When you feel better, do you sometimes stop taking your medicine?, and Sometimes, if you feel worse when you take the medicine, do you stop taking it?. People who answer No/No/No/No are considered high adherents.

Data collection and analysis. The patients were invited to engage in the study during the medical visit and were provided with information about the study; subsequently, their consent to participate in the research was requested. Those who consented were given the data collection instrument to fill it out after the medical visit in the waiting room and return it when finished. The collected information was tabulated in a database in the Excel program and analyzed using the Infostat v/L program. Score calculations corresponding to the domains were performed as previously mentioned. For the descriptive analysis of the numerical variables, the mean, standard deviation, and range were calculated, and for the categorical variables, the absolute and relative frequencies were calculated. The non-parametric Mann-Whitney, Kruskal-Wallis, and Spearman Correlation tests were used for the statistical analysis. The statistical significance value was set at *p*<0.05.

Ethical considerations. The study was approved by the Ethics Committee for Scientific and Technological Research of the Universidad Abierta Interamericana (UAI) under the number 0-1038. The participation of the subjects was voluntary, and the signature of the Informed Consent was requested. The personal data of the participants was always protected, ensuring compliance with the Personal Data Protection Law.

## Results

### Sample characterization

144 subjects take part, mostly male (82.63%), single (61.80%), with university studies (50%), without children (74.30%) and with a steady job (88.19%); of which, 58.33% represented self-employed workers, 26.38% with a dependent employee, and 3.47% with unregistered employment. The mean age was 43.8 years (SD: 11) with a range between 24 and 64 years (Table 1).

**Table 1:** Sociodemographic characteristics**.**

Variable	Frequency	Percent (%)
Gender		
Male	119	82.63
Female	25	17.36
Civil status		
Single	89	61.80
Domestic partnership	29	20.13
Married	19	13.19
Divorced	6	4.16
Widowed	1	0.69
Level of education		
Primary school	5	3.47
Secondary school	32	22.22
Tertiary level	35	24.30
University degree	72	50.00
Employment status		
Employed	127	88.19
Unemployed	17	11.80
Children		
Yes	37	25.69
No	107	74.30

It was found that respondents had a time since HIV infection diagnosis of 9.9 years (SD: 8) with a range between 1 and 31 years. 31.94% presented HIV/AIDS-related complications, being the most common pneumonia (17.35%) and Herpes infection (8.33%). 38.88% were smokers and 43.75% sedentary. The treatment adherence was considered satisfactory (patients compliant with the pharmacological regimen) in 49.30%.

### Psychosocial Adjustment Process

The analysis of Psychosocial Adjustment Process domains showed that Extended family relationship unfolded the higher score (worst perception) with a median of 55.00 (Interquartile range: 20), followed by Health care orientation with a median of 56.00 (IQR: 20), while the lowest score (best perceived) was found in Vocational environment domain with a median of 50.00 (IQR: 12) (Table 2).

**Table 2:** Descriptive statistics of Psychosocial Adjustment to Illness domains

Domain	Mean	Standard deviation	Median	Q1	Q3
Extended Family Relationship	55.98	9.15	55.00	45.00	65.00
Health Care Orientation	55.83	13.95	56.00	45.00	65.00
Sexual Relationship	52.56	8.82	54.00	45.00	59.00
Social Environment	49.99	8.83	50.00	40.00	58.00
Domestic Environment	49.37	9.91	48.00	45.00	53.00
Psychological Distress	49.29	9.16	48.00	40.00	57.00
Vocational Environment	49.03	8.18	50.00	40.00	52.00
Totals	51.72	7.32	51.43	45.71	57.71

In the Health care orientation domain, the item with the lowest score was "Patient expectancies - treatment" with a mean of 0.37, which refers to the different ideas about the treatment or what the patient expects from medical treatment, while "Health care - present disorder" presented the highest score with a mean of 1.33, which shows dissatisfaction or discomfort with the special attention or care that is demanded as part of the treatment.

In the Vocational Environment domain, it was found that the item with the lowest score was "Interpersonal conflicts" with a mean of 0.21, while "Vocational goals" presented the highest score with a mean of 0.65; the above refers to the fact that respondents have largely had to make changes in their work or education-related goals because of their illness although they do not experience relevant conflicts in their workplaces. In the Domestic environment domain, it was found that the item with the lowest score was "Family Communication" with a mean of 0.35, which points out that there has been no marked decrease in communication between the respondent and his/her family members, while "Quality of relations - principal cohabitant” had the highest score with a mean of 0.82.

Regarding the Sexual Relationship domain, the item with the lowest score was "Interpersonal conflict - sexual” with a mean of 0.46 which refers to the constant discussions with the partner about the disease and its impact on sexual relationships, while "Frequency of sexual activity" obtained the highest score with a mean of 1.04 showing the decrease in the frequency of sexual intercourse after diagnosis. In the Extended Family Relationship domain, the lowest scoring item was “Dependency - social” with a mean of 0.29, while "Interest in interacting [with extended family]" was the highest scoring item with a mean of 0.85, showing a marked decrease in interest in interaction with family post-diagnosis.

In the Social Environment domain, the items with the highest mean score were "Family leisure interest" and "Social leisure interest" with a mean of 0.23 in both cases which refer to interest in performing leisure activities with the family or alone from the moment diagnosis was carried out or prior to the time before diagnosis. On the other hand, "Individual leisure activity" obtained the highest score in this domain with a mean of 0.92, and which queries about their current participation in the daily leisure activities individuals performed before diagnosis.

Finally, regarding Psychological Distress, the best-perceived item was "Guilt" with a mean of 0.60 and which refers to the feeling of guilt for things that happen or the feeling of having failed someone, while the item "Anxiety", which exposes the feeling of being tense, fearful, nervous, or anxious, was the highest scored item with a mean of 1.05.

After calculating the raw scores (R-Scores) and converting them to their equivalent T-Score (index from 0 to 100), the corresponding inferential analysis was performed. Regarding age, a negative correlation was found between age and the domains Health care orientation (rs: -0.24, *p*: <0.01), Vocational environment (rs: -0.16, *p*: 0. 05), Domestic environment (rs: -0.19, *p*: 0.02), Extended family relationship (rs: -0.24, *p*: <0.01), Social environment (rs: -0.23, *p*: <0.01) and Psychological distress (rs: -0.21, *p*: 0.01). According to the aforementioned data, it can be firstly inferred that the older the age, the better the psychosocial adjustment process and, secondly, that the young population presents greater psychosocial maladjustment to illness.

Regarding sex, the male population presented a better process of adaptation to the vocational environment (*p*: 0.05) and Extended family relationship (*p*: 0.01).

Regarding employment status, it was found that, in those with steady employment, regardless of contract modality (self-employed, dependent, or unregistered worker), the means of psychosocial adjustment process domains were lower, which point out to a better adaptation process (*p*: <0.01).

When analyzing the relationship between adherence to treatment and the psychosocial adjustment to illness, we found that patients with poor adherence to treatment had the worst adjustment process (see Table 3). These findings were statistically significant, although a cause-effect relationship cannot be established.

**Table 3:** Treatment adherence and Psychosocial Adjustment to Illness domains

PAIS domains	Treatment adherence*	Treatment adherence*	***p*-Value** ^†^
PAIS domains	Good	Poor	***p*-Value** ^†^
Health Care Orientation	50.00 (20.00)	60.00 (20.00)	<0.01
Vocational Environment	45.00 (10.00)	52.00 (12.00)	<0.01
Domestic Environment	45.00 (13.00)	48.00 (15.00)	<0.01
Sexual Relationship	54.00 (14.00)	54.00 (9.00)	0.70
Extended Family Relationship	50.00 (15.00)	60.00 (15.00)	<0.01
Social Environment	40.00 (15.00)	56.00 (14.00)	<0.01
Psychological Distress	47.00 (15.00)	55.00 (14.00)	0.01
Totals	45.71 (6.00)	54.14 (8.14)	<0.01

*Median and Interquartile Range. ^†^Wilcoxon/Mann-Whitney U Test.

Also, was found that the psychosocial adjustment process showed a statistical relationship between physical activity and Sexual relationship domain (*p*: 0.01) and Extended family relationship (*p*:<0.01), while smoking habit displayed a statistical relationship with all psychosocial adjustment domains (*p*: <0.05). In all cases, higher medians were found in those with sedentary as well as smoking habits.

## Discussion

Since the middle of the last century, there has been an exponential increasing interest in the concept of the psychosocial adjustment process in medicine areas inter alia, such as psychiatry, nephrology, oncology, and cardiology. With the advancement of medical sciences, the survival of patients with chronic diseases including HIV infection, has increased, which has demanded the development of coping strategies, maintenance of psychological integrity and, social support, all of which encompass the so-called psychosocial adjustment process.(14,18-19)

For nurses, it is imperative to analyze the processes of coping and adjustment to illness, since imbalances in this area can considerably affect self-care.(18) In this regard, theorists such as Callista Roy and Dorotea Orem have addressed the conceptions of these two aspects, and in an interrelationship between these, several studies have identified a relationship between how the diagnosis is faced and how self-care strategies are implemented.(20,21)

The present study analyzed the process of psychosocial adjustment to illness in people with HIV infection and found an adequate adjustment to the demands of the disease and the relation between that process and self-care, including adherence to pharmacological treatment.

It was found that the patient's relationship with the health care system, as well as the differences between their expectations and the reality of the care process, influenced pharmacological adherence; these findings were consistent with similar studies.(13) Health care professionals should look after patients` health, and in this aspect, the transmission of timely and adequate information along with the maintenance of a cordial relationship based upon mutual trust is considered relevant, since these precepts influence the level of compliance with medical indications.

Concerning employment status, the present study identified an association between having a steady job and adequate psychosocial adjustment in six of the seven domains investigated. Previous studies have identified that having a steady job allows establishing social support, improving self-esteem and the perception of worth, while economic security contributes to survival and a better quality of life, which explains our findings.(22)

Extended family relationship was the domain with the most alterations in the surveyed population, and this was shown to be related to low adherence to pharmacological treatment. These findings are similar to studies in adult and adolescent populations, which described that family cohesion and support, strengthen the ability to cope with external stressors, determine the emotional and behavioral response, and promote treatment compliance as well as self-care.(23,24)

Depression, stress, anxiety, irritability, low self-esteem, and resilience, as well as ineffective coping behaviors such as cognitive avoidance and emotional discharge, are predictors of low adherence to treatment.(25,26) In the present study, the items comprising the psychological distress domain were related to the discontinuation of antiretroviral therapy. It has been suggested that resilience, defined as the ability to transform adversity into opportunities for growth and adaptation, may be important for people with HIV who are susceptible to anxiety and depression, as well as to prevent risk behaviors such as alcohol abuse, smoking, drug addiction, and treatment discontinuity.(27)

The present study constitutes a relevant antecedent due to the lack of national and international bibliography in this area, and could propose new axes of management, treatment, and re-direction of public policy in terms of care for those living with HIV, given the need for a comprehensive approach to this problem and avoiding the biological approach and the pharmacological commercialization of disease. This implies the consideration that people do not get sick alone, but in constant interaction with society, considering aspects such as social relationship (family, friends, work, and education), healthcare orientation, and discrimination as variables of interest for the prevention of the disease; however, to provide appropriate care, interventions are required to improve nurses' attitudes towards people with HIV/AIDS, due to the influence of increasing involvement in training programs and greater efficiency in the provision of quality care.(28)

From nursing, the design of holistic approaching programs, health education, community awareness and the design of new care protocols have proven to bear relevant benefits in other countries in the prevention of complications and higher adherence to self-care indications proposed by health professionals.(29) However, in our country it is necessary to give greater participation to nursing professionals to achieve the task of ensuring the health of this population. Likewise, more studies are required to identify and address the elements that could be influencing adherence to treatment as the basis of self-care for patients with chronic diseases such as HIV infection, in addition to increasing the participation of nursing professionals in specialized primary care programs for this population.

## Conclusion

A high level of discomfort was found with respect to the care required for the treatment of HIV infection (medication, diet, physical activity, and other self-care behaviors), as evidenced by the high mean score on the item "Health care - present disorder" of the Health care orientation domain (mean 1.33), while a lack of conflicts or problems with coworkers due to the disease was identified, as evidenced by the score on the item "Interpersonal conflicts" of the Vocational environment domain (mean 0.21). Male presented higher levels of adjustment and adaptation to the demands from Vocational environment and in Extended family relationships after HIV diagnosis. A statistical significance was identified between adherence to treatment and the psychosocial adjustment process, finding that patients with low adherence to treatment showed inadequate adjustment processes, although no cause-effect relationship can be established for this finding.

This study had limitations such as a small sample size and those results apply to people from middle-income chronic diseases specialized care programs. Data collection carried out by a single researcher and rigorous methodological analysis are strengths of this study. More research is required to deepen the analysis of the relationships between other variables that can affect the process of psychosocial adjustment and pharmacological adherence in HIV patients.
